# Unveiling the phantom: What neuroimaging has taught us about phantom limb pain

**DOI:** 10.1002/brb3.2509

**Published:** 2022-02-26

**Authors:** Jonathan D. Browne, Ryan Fraiser, Yi Cai, Dillon Leung, Albert Leung, Michael Vaninetti

**Affiliations:** ^1^ School of Medicine California University of Science and Medicine Colton California USA; ^2^ Center for Pain Medicine University of California San Diego La Jolla California USA; ^3^ College of Letters and Science University of California Berkeley Berkeley California USA

**Keywords:** DTI, EEG, fMRI, MEG, phantom limb pain

## Abstract

Phantom limb pain (PLP) is a complicated condition with diverse clinical challenges. It consists of pain perception of a previously amputated limb. The exact pain mechanism is disputed and includes mechanisms involving cerebral, peripheral, and spinal origins. Such controversy limits researchers’ and clinicians’ ability to develop consistent therapeutics or management. Neuroimaging is an essential tool that can address this problem. This review explores diffusion tensor imaging, functional magnetic resonance imaging, electroencephalography, and magnetoencephalography in the context of PLP. These imaging modalities have distinct mechanisms, implications, applications, and limitations. Diffusion tensor imaging can outline structural changes and has surgical applications. Functional magnetic resonance imaging captures functional changes with spatial resolution and has therapeutic applications. Electroencephalography and magnetoencephalography can identify functional changes with a strong temporal resolution. Each imaging technique provides a unique perspective and they can be used in concert to reveal the true nature of PLP. Furthermore, researchers can utilize the respective strengths of each neuroimaging technique to support the development of innovative therapies. PLP exemplifies how neuroimaging and clinical management are intricately connected. This review can assist clinicians and researchers seeking a foundation for applications and understanding the limitations of neuroimaging techniques in the context of PLP.

## 1 INTRODUCTION

Pain is unique to individuals who experience it, and the subjective nature of pain challenges its fundamental understanding. Phantom limb pain (PLP) is one such clinical mystery for researchers, clinicians, and patients. While PLP phenomenon has been well documented in recent history, the underlying pathophysiology was poorly understood due to limitation in investigational tools. Ambroise Paré described patients feeling absent limbs following amputations he performed during the 16th century, and in the 17th century, philosopher René Descartes concluded that there might be a dissociation between nerve signals and cognitive interpretation in amputees (Finger & Hustwit, 2003). Later, during the American Civil War, surgeon Silas Weir Mitchell was frustrated by ineffective treatments for PLP and became notable for advocating formal scientific investigations (Finger & Hustwit, 2003). This mystery continues to draw curiosity and interest, and it now leverages modern technology to uncover its truth.

More than merely quenching scientific curiosity, further PLP research is needed to improve the lives of amputees. The amputee population in America is estimated to increase from 1.6 million in 2005 to 3.6 million by 2050 (Ziegler‐Graham et al., 2008). PLP, which is the pain of the amputated limb that is variable in timing, is experienced by up to 79.9% of amputees (Ehde et al., 2000; Ephraim et al., 2005). The severity of phantom limb sensations ranges from nonpainful to disabling and, in some instances, can be physically and psychologically debilitating (Ephraim et al., 2005). For surgical amputees, chronic preoperative pain and acute postoperative phantom pain are risk factors for PLP (Hanley et al., 2007; Larbig et al., 2019). Still, correlations for the severity of pain have been inconsistent (Sherman et al., 1984). These variations in the clinical presentation of PLP continue to burden amputees, warranting a deeper understanding of its mechanism to improve diagnosis and efficacious clinical approaches. As the demand for a conclusive understanding grows, so does the controversy among scientists and clinicians.

PLP is a particularly challenging syndrome to diagnose and treat, which may be related to the fact that, by mechanistic nature, it is challenging to understand. Nonspecific and highly varied symptoms can make diagnosing PLP difficult, requiring a comprehensive history, examination, tests, and exclusion of other possible neuropathies (Ferraro et al., 2016). The variety of PLP therapies, including pharmacologic, cranial stimulation, and sensory therapies, have been inconsistent (Aternali & Katz, 2019; Richardson & Kulkarni, 2017) with some potential being demonstrated among integrative approaches (Subedi & Grossberg, 2011). These challenges parallel the equally complex range of mechanistic explanations of PLP, which involve various combinations of cerebral, spinal, and peripheral nervous system pathologies (Flor et al., 2006). These mechanistic, diagnostic, and management inconsistencies underscore the importance of foundational tools for analyzing PLP. Due to the brain's role in interpreting, processing, and modulating pain, neuroimaging may fulfill this need. In addition to aiding understanding of PLP, neuroimaging may assist the development of PLP therapies.

This article will review current noninvasive imaging modalities for PLP research in mechanistic and therapeutic investigations. It will focus on diffusion tensor imaging (DTI), functional magnetic resonance imaging (fMRI), electroencephalography (EEG), and magnetoencephalography (MEG). Researchers and clinicians utilize each imaging modality in distinct ways to complement dynamic research involving diagnosing, characterizing, and treating PLP. By examining how DTI, fMRI, EEG, and MEG have impacted the understanding of PLP, we aim to summarize a baseline of fundamental imaging techniques to foster further research.

### 1.1 Background on potential pain mechanisms

In order to understand the impact of the major imaging modalities used in this field, it is helpful to briefly review the prevailing discussed potential mechanisms behind PLP. In the properly functioning human nervous system, peripheral noxious stimulus generates a sensation of pain via a cascade of neuronal events. The pathway consists of primary afferent pain fibers which carry the afferent signals from the peripheral nociceptors to the spinal cord where they synapse directly or indirectly via interneuron with the secondary neurons at the dorsal horn of the spinal cord. The afferent signals then ascend via the second‐order neurons to the brain via either spinothalamic or spinoreticular tracts (Steeds, 2009). Passage of this nociceptive information through the brainstem triggers modulatory signals back through the dorsal horn. These modulatory signals alter primary afferent neuron propagation which can facilitate or inhibit further peripheral nociceptive information (Renn & Dorsey, 2005). Additionally, supraspinal pain signal processing and modulation are important for healthy pain perception. First, the thalamus and pons relate afferent sensory signals to other supraspinal regions. Other supraspinal groups include the somatosensory cortices and inferior parietal lobe, anterior cingulate cortex (ACC) and insula (IN), and dorsolateral prefrontal cortex, which are also important sensory discriminatory, affective, and modulatory regions, respectively (Leung, 2020). Furthermore, supraspinal modulatory functional connectivity deficits have been associated with white matter tract deficits, emphasizing the vital role of supraspinal processing (Leung et al., 2016, 2018). These normal pain mechanisms involve the intricate relationship between peripheral, spinal, and supraspinal regions.

PLP draws attention because there is still no consensus on its mechanism. Cerebral, spinal, and peripheral explanations each bear scientific evidence, perpetuating the controversy (Collins et al., 2018). Mechanisms within these groups are not mutually exclusive and PLP may be explained by some combination. Furthermore, researchers speculate if PLP may be a cluster of pain disorders, rather than a single disorder (Griffin & Tsao, 2014). Researchers have prioritized this mechanistic puzzle as it is essential for providing quality care to these patients.

Cerebral mechanisms consist of cortical reorganization, alterations in sensory and motor feedback, and pain memory (Flor et al., 2006). PLP is commonly correlated with reorganization and furthermore related to the self‐perception of one's own body (Subedi and Grossberg, 2011). In support of cerebral mechanisms, a 1998 study found hemispheric differences in cortical representation in traumatic amputees absent in subjects with congenital absence of limb (Montoya et al., 1998).

Spinal mechanisms relate to amputation‐related nerve injury causing spinal cord hypersensitization and further reorganization of spinal cord areas formerly occupied by functioning afferent nerves (Flor et al., 2006). Connections between the proximal sections of amputated nerves can form disruptive connections with receptive spinal nerves. Additionally, distorted neuronal activity, hyperexcitability, and central nociceptive neuron firing pattern changes may also contribute to PLP (Subedi and Grossberg, 2011).

The peripheral mechanisms involve nerve ending and dorsal root ganglion reorganization following amputations (Flor et al., 2006). Efficacious pre‐ and postoperative peripheral interventions for PLP support this explanation. Patients receiving peripheral nerve interfaces before surgery have had lower rates of peripheral neuromas and PLP (Kubiak et al., 2019). Additionally, minimally invasive percutaneous peripheral nerve stimulation programs improved functionality in patients with chronic pain postamputation (Gilmore et al., 2019). Peripheral nervous system treatment has addressed PLP functionality and pain, which validates this mechanism.

Cerebral, spinal, and peripheral PLP mechanisms have each endured scientific evaluation with no distinct victor. These are also not mutually exclusive and PLP may be a product of a combination of these mechanisms. Makin and Flor further expand upon the multifactorial nature through a review of factors beyond remapping that may come together to contribute to PLP (Makin & Flor, 2020). Broad consideration of mechanism and dynamic changes warrants a comprehensive analysis of this complex disease. Investigators continue to explore this scientific question using several specialized neuroimaging techniques.

## 2 METHODS

A literature search was conducted using the PubMed database between January 2020 and August 2021. The literature search was organized using the following keywords/keyword combinations: “phantom limb pain and diffusion tensor imaging (DTI),” “phantom limb pain mechanism,” “phantom limb pain and electroencephalography (EEG),” “phantom limb pain and functional magnetic resonance imaging (fMRI),” “phantom limb pain and amputation,” “phantom pain,” “phantom limb pain and mirror therapy,” “phantom limb pain and magnetoencephalography (MEG),” “phantom limb pain and therapeutics,” “diffusion tensor imaging (DTI),” “electroencephalography (EEG),” “magnetoencephalography (MEG),” “functional magnetic resonance imaging (fMRI).” The articles generated from the search were then screened and additional articles referenced by the searched articles were also utilized. Articles were selected based on the inclusion of amputees with PLP or phantom sensations along with the utilization of DTI, fMRI, EEG, or MEG to investigate mechanism or response to therapy.

## 3 RESULTS

### 3.1 Diffusion tensor imaging

DTI is a variant of conventional MRI that has become a standard tool in researching PLP. As a general MRI principle, tissue microstructure determines water diffusion, which translates into an image. Anisotropy describes water diffusion that is directionally dependent while isotropy describes unrestricted water diffusion; white matter is more anisotropic than gray matter, while cerebrospinal fluid is isotropic (Hagmann et al., 2006; Pierpaoli et al., 1996). DTI capitalizes on white matter tracts to assess structural integrity and connectivity (Bandettini, 2009). In PLP, DTI has become the most common tool for evaluating anatomical changes.

Important DTI scalars include axial diffusivity (AD), radial diffusivity (RD), mean diffusivity (MD), and fractional anisotropy (FA). AD and RD characterize rates of diffusion in principal and perpendicular directions, respectively, while MD is the net displacement of water molecules (Feldman et al., 2010). FA is a ratio that describes the degree of anisotropic diffusion (Feldman et al., 2010). These scalars allow DTI to interpret structural changes within the brain. A 2019 study employed DTI to determine a connection between PLP and white matter changes. Interestingly, these researchers found symmetrically increased white matter AD bilaterally, but a stronger white matter RD association with visual analog scale (VAS) score in the corpus callosum and hemisphere associated with the amputated limb (Seo et al., 2019). Guo et al. (2019) studied changes in FA following upper‐limb amputation using DTI and positively correlated contralateral middle temporal gyrus nodal strength with the magnitude of PLP. In contrast, Jiang et al. studied lower‐limb amputees using DTI and described ipsilateral decreased FA in the superior corona radiata, sub‐temporal lobe white matter, and inferior fronto‐occipital fasciculus. Additionally, they noted contralateral reduced FA in the left premotor cortex. Utilizing tractography in the premotor cortices, they also found altered interhemispheric fibers (Jiang et al., 2015).

Structural analysis is useful for understanding physical changes due to PLP and potentially planning for interventions. The properties of DTI have propelled it to become a standard tool for such structural analysis. Corpus callosum changes identified via DTI provide clues regarding the connection between phantom sensations and sensorimotor cortex inhibition (Simões et al., 2012). Furthermore, Owen et al. (2007) utilized DTI tractography to guide deep brain stimulation in an amputee experiencing stump pain. DTI applications and key findings are summarized in Table 1.

**TABLE 1:** Diffusion tensor imaging (DTI) and phantom limb pain (PLP)

Author	Sample size: Study control	Application	Key findings
Seo et al., 2019	10 16	Identified structural changes	Stronger white matter radial diffusivity in corpus callosum and hemisphere associated with amputated limb
Guo et al., 2019	22 15	Identified structural changes	Positive correlation between contralateral middle temporal gyrus nodal strength and PLP magnitude
Jiang et al., 2015	17 18	Identified structural changes	Decreased ipsilateral fractional anisotropy in superior corona radiata, sub‐temporal lobe white matter, and inferior fronto‐occipital fasciculus
Simões et al., 2012	9^a^ 9	Identified structural changes	Structural changes in corpus callosum; related “painless” phantom sensations and sensorimotor cortex inhibition
Owen et al., 2007	1 0	Preoperative deep brain stimulation planning	Utility of DTI in assisting planning for PLP interventions

*Note*: Study sample size reflects amputees with phantom limb pain unless otherwise noted.

^a^Amputees with “painless” phantom sensations.

Despite its growing prevalence in neuroimaging, DTI maintains technical issues such as subject motion, eddy currents, and low resolution (Bandettini, 2009). A review of DTI imaging by Alexander et al. found that its measure of FA was sensitive for finding microstructure changes, but this alone was less useful for characterizing such changes. They emphasized the importance of utilizing additional DTI scalars in concert for comprehensive cerebral pathology classification (Alexander et al., 2007). Hakulinen et al. caution the acceptance of FA, considering it to be nonspecific to various pathologies. They also note the variation in the DTI technique, potentially compromising different reports’ comparability without a validated method. The review concludes that the circular method has better repeatability, while the freehand method has less variation; these characteristics may be advantageous for studying distinct aspects of the brain (Hakulinen et al., 2012). Furthermore, Soares et al. (2013) address the technical components of DTI interpretation at each stage of data collection and propose conformity that may serve to reduce variability among researchers.

**FIGURE 1:**
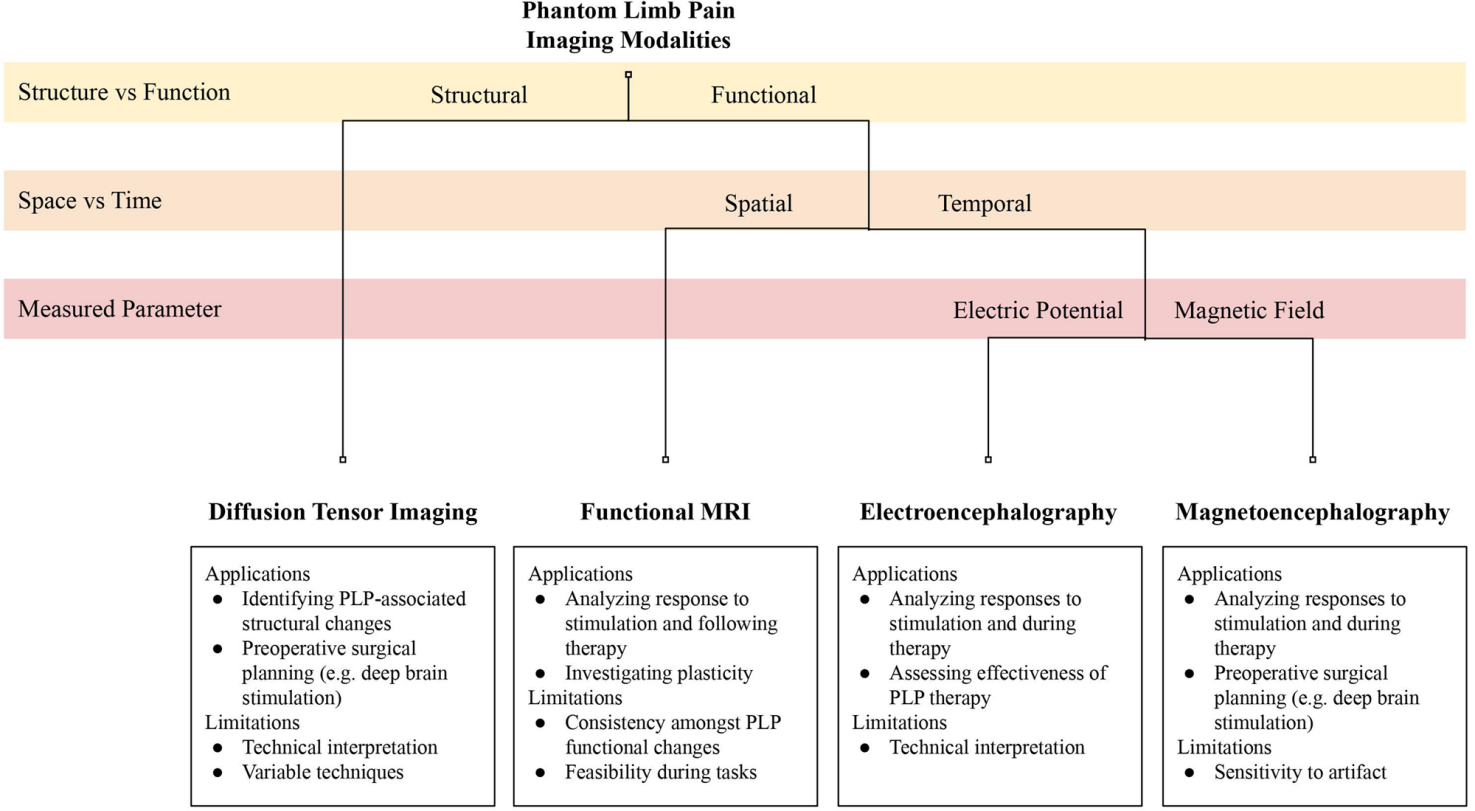
Flowchart of imaging modalities within the context of phantom limb pain.

In summary, DTI exploits water diffusion due to tissue microstructures to reveal critical structural changes due to PLP. As depicted in Figure 1, analysis of these structural changes can contribute to studying cerebral mechanisms of PLP. Technical aspects limit this imaging technique and may compromise data collection and interpretation. DTI should continue to be used to characterize how particular structural changes relate to the presence and severity of PLP in correlation with functional changes.

### 3.2 Functional magnetic resonance imaging

fMRI employs many of the same concepts as DTI. However, in contrast to DTI structural imaging, fMRI is an essential functional imaging technique utilized in PLP research. Magnetic forces create particular arrangements of water molecules. Additionally, oxyhemoglobin and deoxyhemoglobin have different magnetic properties (Bunge & Kahn, 2009). Thus, fMRI can measure tissue perfusion and changes in oxygen that are interpreted to create a functional activity map (Logothetis, 2008). It is a commonly used neuroimaging technique due to logistic factors such as availability, low cost, and low risks (Bunge and Kahn, 2009). Compared to the other functional imaging techniques discussed in this review (EEG and MEG), fMRI has more substantial spatial resolution but lower temporal resolution (Meyer‐Lindenberg, 2010).

Simoes et al. (2012) combined fMRI and DTI to study cortical and colossal plasticity and found that neuroplastic modifications were present in subjects who reported PLP and those who reported only phantom limb sensations . Pasaye et al. (2010) also utilized fMRI to show activation of distinct areas within the brain upon stump stimulation. Andoh et al. (2017) identified inter‐ and intrahemispheric differences in amputees via fMRI in addition to bilateral SI and intraparietal sulcus activation upon phantom sensation evocation. In a later study, Andoh et al. (2020) also utilized fMRI during a virtual reality (VR) task to demonstrate that motor cortex activity was positively related to PLP intensity.

When characterizing the efficacy of PLP rehabilitative techniques, researchers often utilize fMRI. Foell et al. focused on fMRI to identify physical changes in response to mirror therapy, which involved movement of the intact limb in front of a mirror to create the perception of movement in the amputated limb. They report decreased inferior parietal cortex activity and the reversal of maladaptive cortical reorganization (Foell et al., 2014). In a case report about using chronic motor cortex stimulation to treat PLP, researchers used fMRI for precise surgical electrode placement and monitored the patient's response to therapy (Roux et al., 2008). A study of 13 upper limb amputees utilized fMRI that showed reduced cortical reorganization in PLP patients following mental imagery therapy by contrasting pretraining diffuse cortical activation upon motor tasks, such as a lip purse, with post‐training isolated lip area activation. This 6‐week training additionally correlated reductions in pain intensity with a decrease in cortical reorganization (MacIver et al., 2008). fMRI applications and key findings are summarized in Table 2.

**TABLE 2:** Functional magnetic resonance imaging (fMRI) and phantom limb pain (PLP)

Author	Sample size: Study control	Application	Key findings
Simões et al., 2012	9^a^ 9	Response to phantom limb stimulation	Functional remapping of S1 in setting of “painless” phantom sensations
Pasaye et al., 2010	2 6	Response to phantom limb stimulation	Distinct Brodmann areas activated following stump stimulation
Andoh et al., 2017	5 5	Response to phantom limb stimulation	Hemispheric differences in amputees with “painless” phantom sensations; bilateral SI and intraparietal sulcus activation
Andoh et al., 2020	40^b^ 20	During virtual reality therapy	Motor cortex activity positively related to PLP intensity
Foell et al., 2014	11 0	Response to mirror therapy	Decreased inferior parietal cortex activity and reversal of maladaptive cortical reorganization
Roux et al., 2008	1 0	Assisted surgical electrode placement and monitored response to motor cortex stimulation	Detected inhibiting effects on primary sensorimotor cortex and contralateral primary motor and sensitive cortices
MacIver et al., 2008	13 6	Response to mental imagery therapy	Reduced cortical reorganization, which correlated with reduction in pain intensity

*Note*: Study sample size reflects amputees with phantom limb pain unless otherwise noted.

^a^ Amputees with “painless” phantom sensations.

^b^ Compared 20 PLP amputees, 20 non‐PLP amputees, and 20 controls.

Logothetis explains that fMRI interpretation requires caution as this neuroimaging technique may not make key distinctions, such as top‐down versus bottom‐up, excitation versus inhibition, or regional differences (Logothetis, 2008). Another recent review of 10 fMRI investigations from 2001 to 2015 found that this imaging technique did not comprehensively support maladaptive brain plasticity, including the relationship between pain intensity and reorganization (Jutzeler et al., 2015). In studying fMRI during a VR movement task, Andoh et al. (2020) found that fMRI findings for PLP may vary based on methodology. These findings suggest that fMRI is inconsistent in evaluating changes due to PLP, or perhaps there are gaps in understanding of plasticity in PLP. This principle reiterates the importance of the link between PLP and neurologic adaptation and the need for collaborative techniques.

fMRI measures functional changes with strong spatial resolution but is prone to certain ambiguities in interpretation. The lack of critical distinctions may be why fMRI studies have shown mixed results in PLP research. fMRI has utility in supplementing studies of PLP therapies and interventions (Figure 1).

### 3.3 Electroencephalography

EEG is a temporal‐functional imaging technique that is useful in PLP research. EEG interprets electrical flow across membranes as neurons depolarize. It is distinct from the other imaging techniques discussed in this review by recording real‐time measurements in varying cognitive states (Bunge and Kahn, 2009). By measuring perpendicular electrical flow, EEG can analyze gyri and deep sulci pyramidal cells (Bunge and Kahn, 2009). EEG has been further touted, along with MEG, as a method of analyzing cortical reorganization due to advantageous temporal and spatial resolution (Wiech et al., 2004). Other researchers have challenged EEG and MEG for truly assessing signal sources, and suggest employing fMRI as a tertiary, complementary component for signal localization (Bunge and Kahn, 2009; Cottereau et al., 2015). EEG, along with MEG, has higher temporal resolution but lower spatial resolution than fMRI (Meyer‐Lindenberg, 2010). The unique ability to perform EEG simultaneously with fMRI further distinguishes this tool as a select method for capturing nonrepeatable events (Bandettini, 2009).

A case report of a subject with a congenitally absent limb found EEG signatures during attempted movements of the phantom limb to be similar to a cohort of healthy volunteers (Walsh et al., 2015). Another study analyzed a cohort of 22 right‐hand amputees via EEG showed distinct global and local network changes in alpha and beta bands (Lyu et al., 2016). An investigation of the connection between pain catastrophizing and PLP using EEG showed that these patients had an increased response at the N/P135 dipole of the affected side, suggesting that attention to stimuli may be associated with PLP (Vase et al., 2012).

Mirror therapy has been studied as a potential PLP treatment, but recent developments in VR have enabled inventive therapeutic techniques. One such VR investigation utilized EEG and observed PLP alleviation and alpha wave coherence during stimulation of referred sensation areas (Osumi et al., 2020). EEG presents a safe and practical way to monitor the forefront of therapeutic techniques for PLP. It allows researchers to gather robust functional change data during therapies. EEG applications and key findings are summarized in Table 3.

**TABLE 3:** Electroencephalography (EEG) and phantom limb pain (PLP)

Author	Sample size: Study control	Application	Key findings
Walsh et al., 2015	1 0	During attempted movement of phantom limb	Left frontal EEG pattern similar to a healthy cohort of a previous report
Lyu et al., 2016	22 24	Assessed reorganization following amputation	Distinct global and local network changes in alpha and beta bands
Vase et al., 2012	18 0	Response to phantom limb stimulation	Increased response at the N/P135 dispose of the affected side; attention to stimuli may be associated with PLP
Osumi et al., 2020	2 0	During virtual reality rehabilitation with vibrotactile stimulation	PLP alleviation and increased alpha wave coherence

*Note*: Study sample size reflects amputees with phantom limb pain unless otherwise noted.

While it has many applications and strengths, EEG is limited by lower spatial resolution than fMRI (Meyer‐Lindenberg, 2010). The spatial resolution has important utility in the investigation of cerebral PLP mechanisms. If only certain superficial regions are reliably captured, deeper cortical reorganization may be missed. Additionally, a review of the EEG technique concluded that EEG deflections are challenging to interpret, and this tool should be one of many used in conjunction (Jackson & Bolger, 2014). EEG alone therefore may not provide adequate information about functional changes due to PLP.

EEG is one of the two main techniques for identifying functional changes with temporal resolution. It measures changes in electrical potential perpendicular to the direction of neuronal signal propagation. While EEG is limited by a lack of spatial resolution, its inherent design allows it to be easily used alongside other tools to provide comprehensive results. EEG is a practical way to monitor functional changes while developing PLP therapies (Figure 1).

### 3.4 Magnetoencephalography

MEG is another temporal‐functional imaging technique used in PLP research. It functions by measuring the small magnetic fields created by electrical currents involved in neuronal signaling. The measured magnetic dipole is 90° off phase with the electrical one. The electrical and magnetic fields detected by EEG and MEG are generated by extracellular and intracellular currents, respectively (Singh, 2014). Both of these measured phenomena occur at directions perpendicular to that of neuronal signal propagation. Because it assesses magnetic activity at and parallel to the brain's surface, MEG is limited to the analysis of superficial sulci pyramidal cells (Bunge & Kahn, 2009). As mentioned before, EEG and MEG share a common caveat as they both have difficulty localizing signal sources (Bunge & Kahn, 2009). MEG also has a higher temporal resolution but lower spatial resolution compared to fMRI (Meyer‐Lindenberg, 2010).

In a 2001 study, researchers induced acute left thenar pain in healthy non‐PLP patients through capsaicin injections. MEG analysis revealed increased proximity between hand and lip representation, suggesting an acute reorganization in response to the stimulus (Sörös et al., 2001). Blume et al. later utilized MEG and identified lip and hand cortical reorganization following an amputated limb replantation. In contrast to other reports, they also found a negative correlation between pain and cortical reorganization (Blume et al., 2014).

Kringelbach et al. employed MEG to investigate the effect of deep brain stimulation on a PLP patient. The researchers found changes in mid‐anterior orbitofrontal and subgenual cingulate activity after stimulation was stopped and associated these regions of the brain with pain relief. Their results demonstrate that MEG is useful for identifying response to therapy and potential surgical targets for pain relief (Kringelbach et al., 2007). Another investigation of brain–machine interface training integrated MEG reading with a robotic hand. Interestingly, they found this training to intensify pain when used with the phantom limb. At the same time, it reduced pain during dissociative prosthetic‐phantom hand training, further suggesting a link between plasticity and pain (Yanagisawa et al., 2016). MEG applications and key findings are summarized in Table 4.

**TABLE 4:** Magnetoencephalography (MEG) and phantom limb pain (PLP)

Author	Sample size: Study control	Application	Key findings
Blume et al., 2014	13 0	Assessed reorganization following amputation	Negative correlation between pain and cortical reorganization
Kringelbach et al., 2007	1 0	Response to deep brain stimulation and identified surgical targets	Return of pain associated with changes in mid‐anterior orbitofrontal and subgenual cingulate activity after stopping stimulation
Yanagisawa et al., 2018	10^a^ 0	During brain‐machine interface training	MEG‐based therapy can induce cortical plasticity to help treat PLP

*Note*: Study sample size reflects amputees with phantom limb pain unless otherwise noted.

^a^ Assessed one amputee and nine brachial plexus avulsion patients with phantom pain.

Despite its usefulness and safety, MEG has sensitivity to artifacts. Ray et al. addressed the challenge of deep brain stimulation artifact when using MEG by focusing on the occipital lobe following a visual stimulus (Ray et al., 2009). The study shows that this tool can provide relevant information if researchers account for its limitations.

MEG is another primary technique for identifying functional changes with temporal resolution. In contrast to EEG, MEG detects magnetic activity parallel to the brain surface. This imaging modality is mostly limited by potential artifacts, which an adapted approach may control. MEG has promising future use for studying robotic and interventional therapy in PLP research (Figure 1).

## 4 DISCUSSION

Neuroimaging has proven paramount in the study of PLP (Figure 1). DTI readily outlines structural changes and has potential for surgical applications but is frequently cited for technical limitations, such as subject motion and resolution. Additionally, DTI has often been criticized for variation in measuring technique and data interpretation. fMRI captures functional changes with spatial resolution in various PLP therapies, but cannot make critical neurologic distinctions, which limits data interpretation without behavioral or structural correlation. EEG and MEG are notable for identifying functional changes with a strong temporal resolution and are differentiated by perpendicular electric and parallel magnetic activity, respectively. EEG is significant for spatial limitations, while both EEG and MEG are limited by artifact. Overall, all four imaging techniques provide unique perspectives that have shaped the modern understanding of PLP.

Accessibility and practicality are common barriers that limit PLP neuroimaging. The study scale is often resource dependent, which has restricted how much imaging data can be collected. Limited reproducibility of neuroimaging findings may also hinder the analysis of PLP in certain cases. Consistent techniques and collaboration may alleviate the burden on groups studying PLP. Additionally, the automation of imaging analysis using artificial intelligence and machine learning algorithms may generate uniformity among data interpretation (Hu et al., 2019; Vieira et al., 2017). These advancements enable the synthesis of data sets to help map neural changes. Robust data collection illustrates the key intersection of imaging and analytical technology, especially in the context of clinical disease. As this field evolves, researchers will continue to utilize neuroimaging aiming to provide fundamental insight into PLP's pathogenesis and treatment.

## FUNDING INFORMATION

None.

## CONFLICT OF INTEREST

The authors declare no conflict of interest.

### PEER REVIEW

The peer review history for this article is available at https://publons.com/publon/10.1002/brb3.2509
